# Supplementary material for: Ultra-deep sequencing validates safety of CRISPR/Cas9 genome editing in human hematopoietic stem and progenitor cells
Source: Nat Commun. 2022 Aug 11;13:4724. doi: 10.1038/s41467-022-32233-z (PMC9372057; doi:10.1038/s41467-022-32233-z)
Supplement: Supplementary file 1 — Supplementary Information [file 41467_2022_32233_MOESM1_ESM.pptx]

## Slide 1
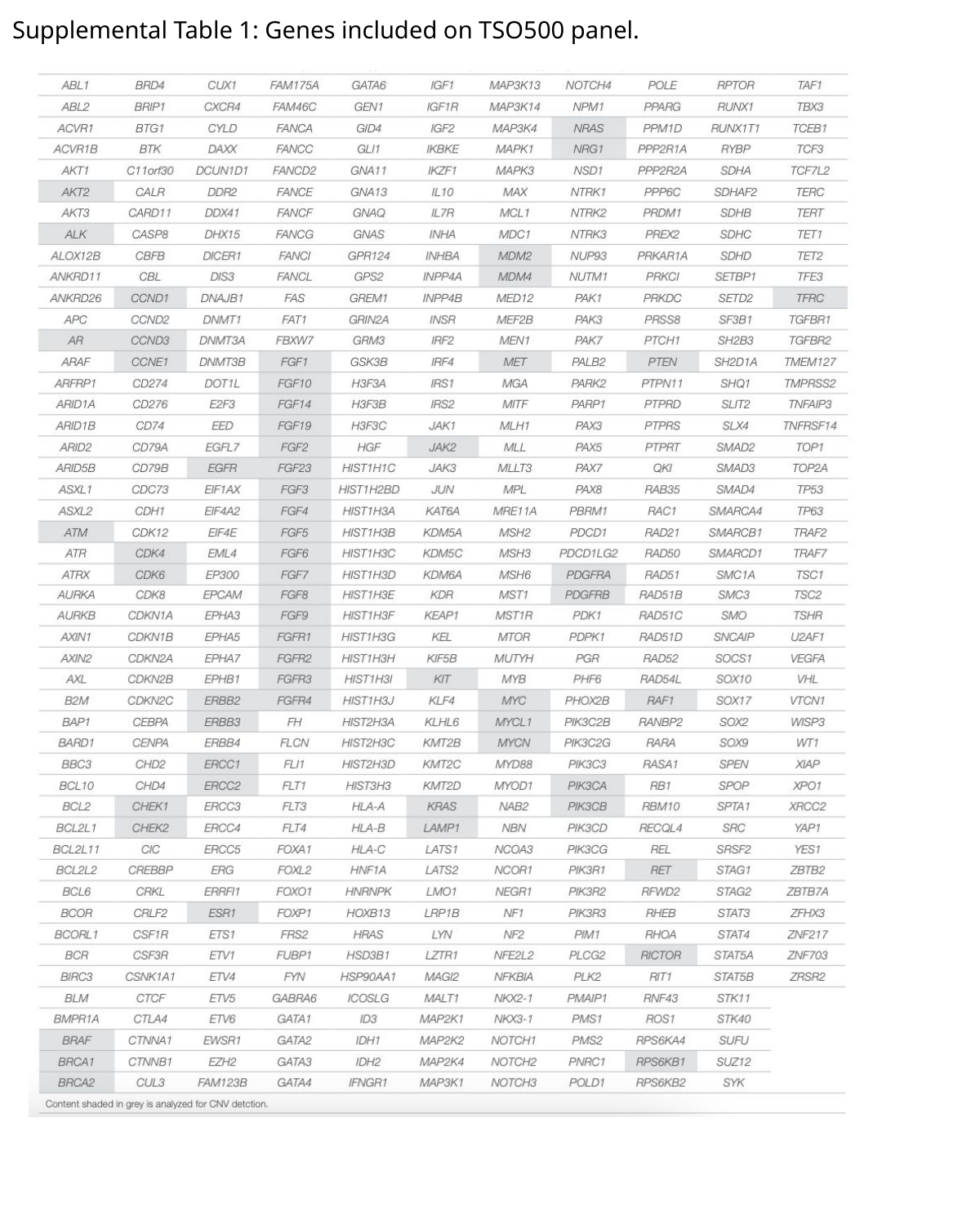

Supplemental Table 1: Genes included on TSO500 panel.

## Slide 2
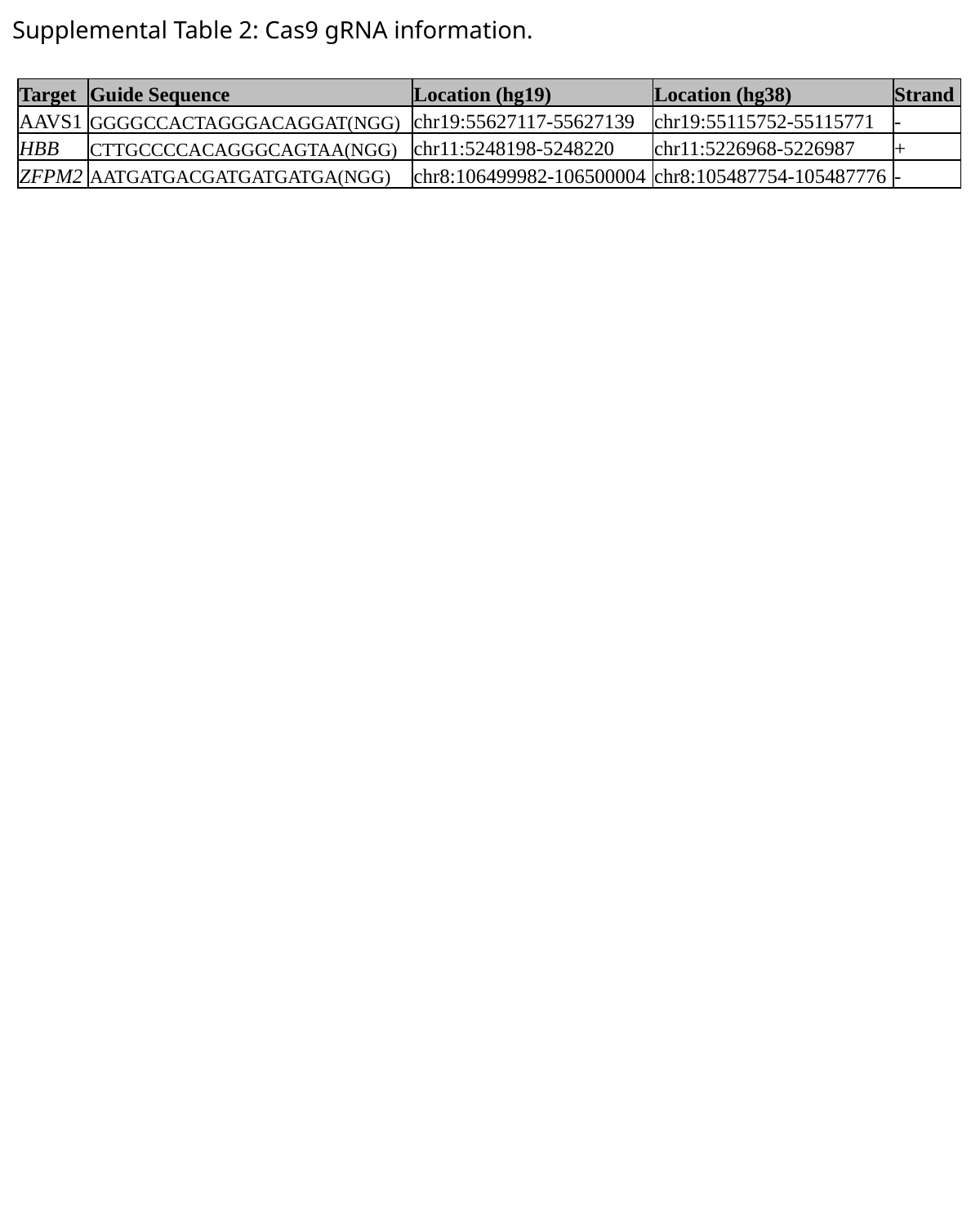

Supplemental Table 2: Cas9 gRNA information.
| Target | Guide Sequence | Location (hg19) | Location (hg38) | Strand |
| --- | --- | --- | --- | --- |
| AAVS1 | GGGGCCACTAGGGACAGGAT(NGG) | chr19:55627117-55627139 | chr19:55115752-55115771 | - |
| HBB | CTTGCCCCACAGGGCAGTAA(NGG) | chr11:5248198-5248220 | chr11:5226968-5226987 | + |
| ZFPM2 | AATGATGACGATGATGATGA(NGG) | chr8:106499982-106500004 | chr8:105487754-105487776 | - |

## Slide 3
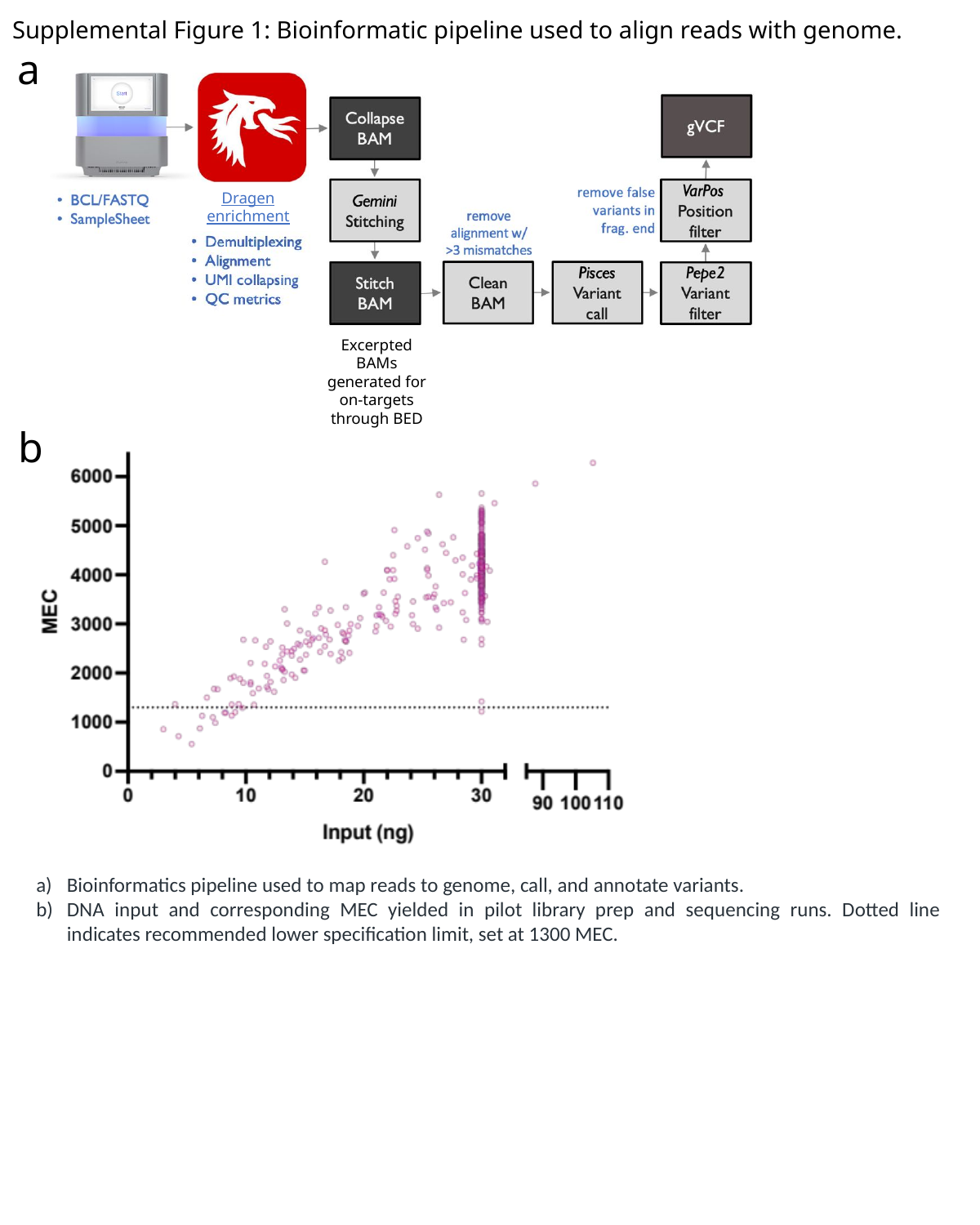

Supplemental Figure 1: Bioinformatic pipeline used to align reads with genome.
a
Dragen enrichment
Excerpted BAMs generated for on-targets through BED
b
Bioinformatics pipeline used to map reads to genome, call, and annotate variants.
DNA input and corresponding MEC yielded in pilot library prep and sequencing runs. Dotted line indicates recommended lower specification limit, set at 1300 MEC.

## Slide 4
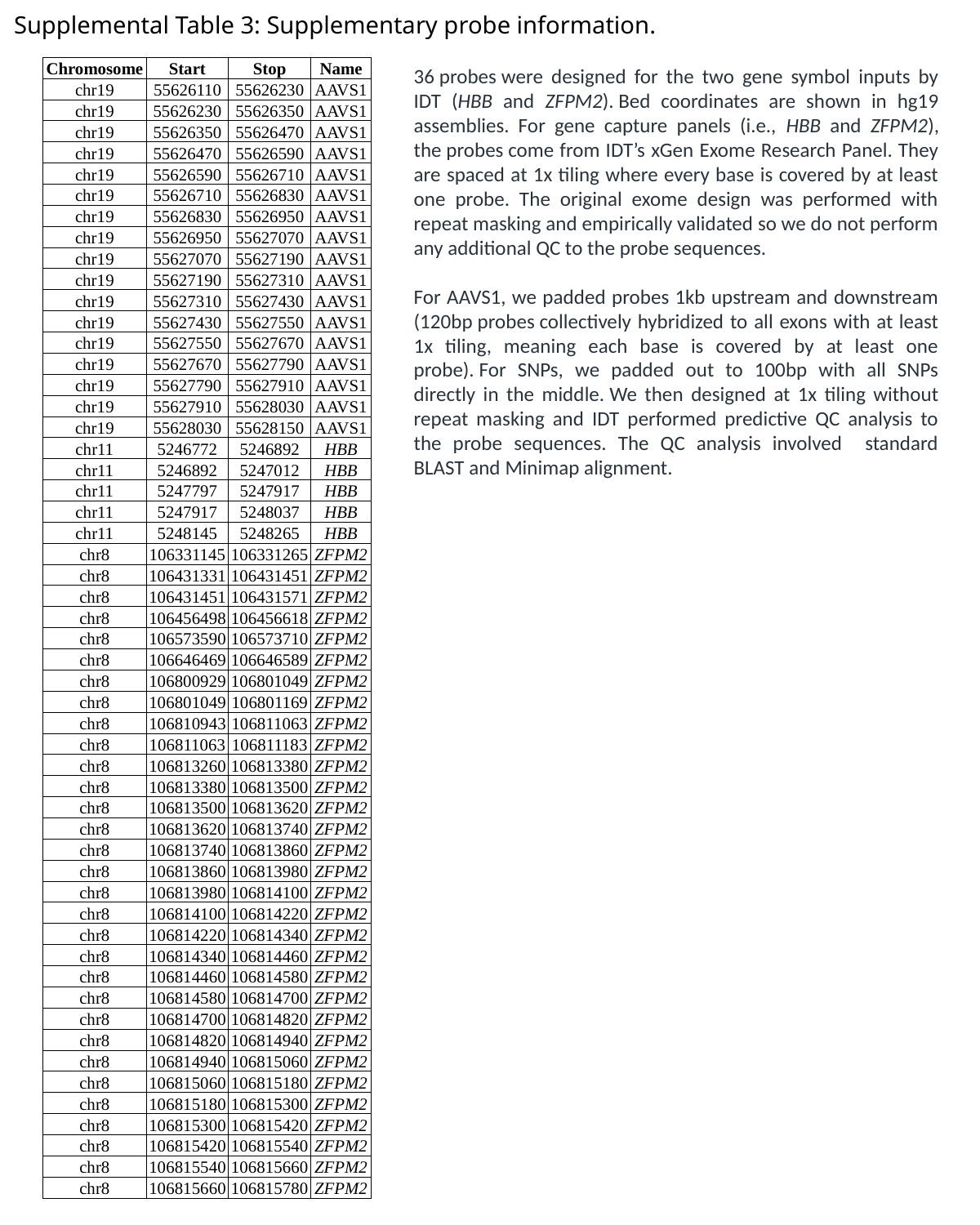

Supplemental Table 3: Supplementary probe information.
| Chromosome | Start | Stop | Name |
| --- | --- | --- | --- |
| chr19 | 55626110 | 55626230 | AAVS1 |
| chr19 | 55626230 | 55626350 | AAVS1 |
| chr19 | 55626350 | 55626470 | AAVS1 |
| chr19 | 55626470 | 55626590 | AAVS1 |
| chr19 | 55626590 | 55626710 | AAVS1 |
| chr19 | 55626710 | 55626830 | AAVS1 |
| chr19 | 55626830 | 55626950 | AAVS1 |
| chr19 | 55626950 | 55627070 | AAVS1 |
| chr19 | 55627070 | 55627190 | AAVS1 |
| chr19 | 55627190 | 55627310 | AAVS1 |
| chr19 | 55627310 | 55627430 | AAVS1 |
| chr19 | 55627430 | 55627550 | AAVS1 |
| chr19 | 55627550 | 55627670 | AAVS1 |
| chr19 | 55627670 | 55627790 | AAVS1 |
| chr19 | 55627790 | 55627910 | AAVS1 |
| chr19 | 55627910 | 55628030 | AAVS1 |
| chr19 | 55628030 | 55628150 | AAVS1 |
| chr11 | 5246772 | 5246892 | HBB |
| chr11 | 5246892 | 5247012 | HBB |
| chr11 | 5247797 | 5247917 | HBB |
| chr11 | 5247917 | 5248037 | HBB |
| chr11 | 5248145 | 5248265 | HBB |
| chr8 | 106331145 | 106331265 | ZFPM2 |
| chr8 | 106431331 | 106431451 | ZFPM2 |
| chr8 | 106431451 | 106431571 | ZFPM2 |
| chr8 | 106456498 | 106456618 | ZFPM2 |
| chr8 | 106573590 | 106573710 | ZFPM2 |
| chr8 | 106646469 | 106646589 | ZFPM2 |
| chr8 | 106800929 | 106801049 | ZFPM2 |
| chr8 | 106801049 | 106801169 | ZFPM2 |
| chr8 | 106810943 | 106811063 | ZFPM2 |
| chr8 | 106811063 | 106811183 | ZFPM2 |
| chr8 | 106813260 | 106813380 | ZFPM2 |
| chr8 | 106813380 | 106813500 | ZFPM2 |
| chr8 | 106813500 | 106813620 | ZFPM2 |
| chr8 | 106813620 | 106813740 | ZFPM2 |
| chr8 | 106813740 | 106813860 | ZFPM2 |
| chr8 | 106813860 | 106813980 | ZFPM2 |
| chr8 | 106813980 | 106814100 | ZFPM2 |
| chr8 | 106814100 | 106814220 | ZFPM2 |
| chr8 | 106814220 | 106814340 | ZFPM2 |
| chr8 | 106814340 | 106814460 | ZFPM2 |
| chr8 | 106814460 | 106814580 | ZFPM2 |
| chr8 | 106814580 | 106814700 | ZFPM2 |
| chr8 | 106814700 | 106814820 | ZFPM2 |
| chr8 | 106814820 | 106814940 | ZFPM2 |
| chr8 | 106814940 | 106815060 | ZFPM2 |
| chr8 | 106815060 | 106815180 | ZFPM2 |
| chr8 | 106815180 | 106815300 | ZFPM2 |
| chr8 | 106815300 | 106815420 | ZFPM2 |
| chr8 | 106815420 | 106815540 | ZFPM2 |
| chr8 | 106815540 | 106815660 | ZFPM2 |
| chr8 | 106815660 | 106815780 | ZFPM2 |
36 probes were designed for the two gene symbol inputs by IDT (HBB and ZFPM2). Bed coordinates are shown in hg19 assemblies. For gene capture panels (i.e., HBB and ZFPM2), the probes come from IDT’s xGen Exome Research Panel. They are spaced at 1x tiling where every base is covered by at least one probe. The original exome design was performed with repeat masking and empirically validated so we do not perform any additional QC to the probe sequences.
For AAVS1, we padded probes 1kb upstream and downstream (120bp probes collectively hybridized to all exons with at least 1x tiling, meaning each base is covered by at least one probe). For SNPs, we padded out to 100bp with all SNPs directly in the middle. We then designed at 1x tiling without repeat masking and IDT performed predictive QC analysis to the probe sequences. The QC analysis involved standard BLAST and Minimap alignment.

## Slide 5
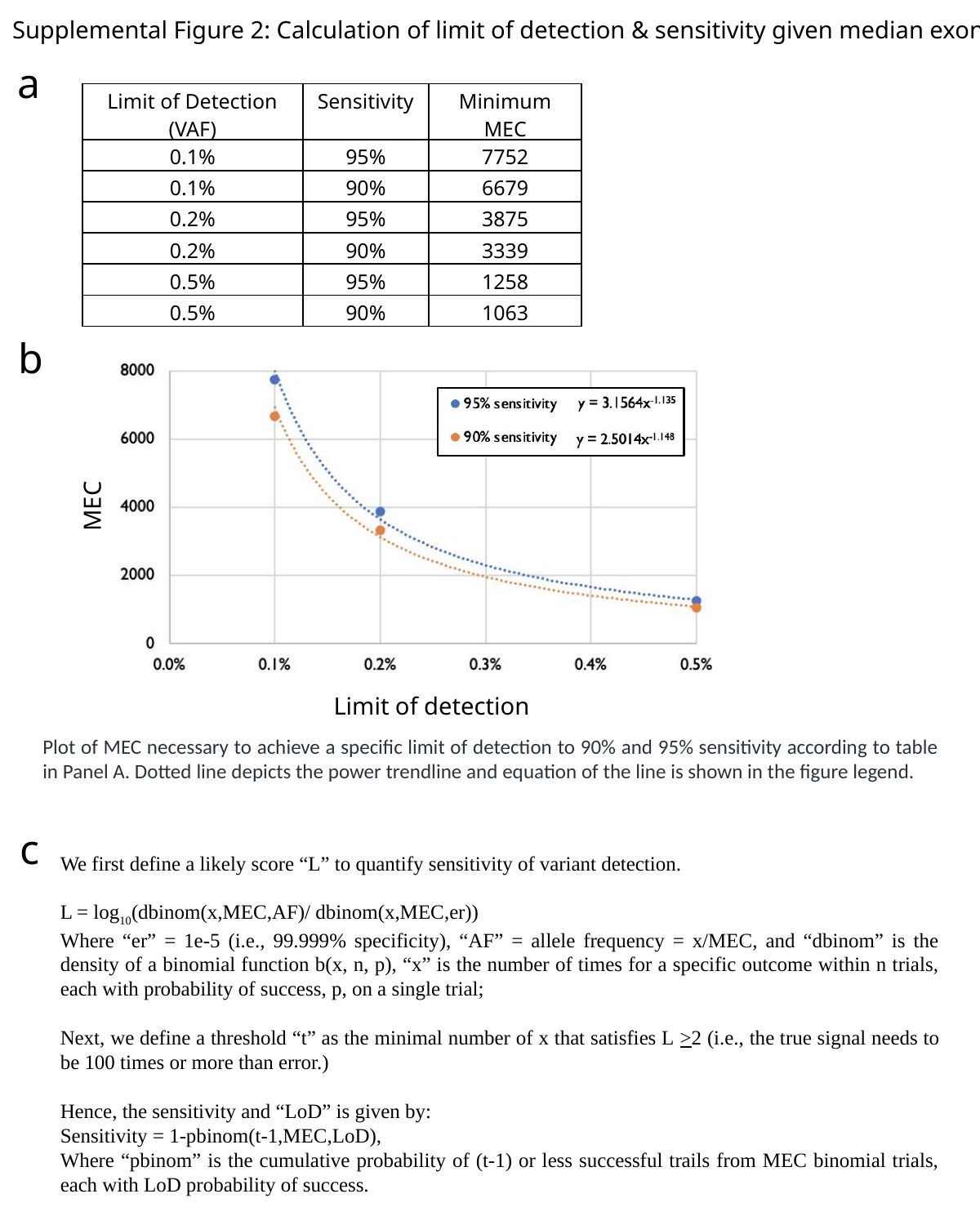

Supplemental Figure 2: Calculation of limit of detection & sensitivity given median exon coverage.
a
| Limit of Detection (VAF) | Sensitivity | Minimum MEC |
| --- | --- | --- |
| 0.1% | 95% | 7752 |
| 0.1% | 90% | 6679 |
| 0.2% | 95% | 3875 |
| 0.2% | 90% | 3339 |
| 0.5% | 95% | 1258 |
| 0.5% | 90% | 1063 |
b
MEC
Limit of detection
Plot of MEC necessary to achieve a specific limit of detection to 90% and 95% sensitivity according to table in Panel A. Dotted line depicts the power trendline and equation of the line is shown in the figure legend.
c
We first define a likely score “L” to quantify sensitivity of variant detection.
L = log10(dbinom(x,MEC,AF)/ dbinom(x,MEC,er))
Where “er” = 1e-5 (i.e., 99.999% specificity), “AF” = allele frequency = x/MEC, and “dbinom” is the density of a binomial function b(x, n, p), “x” is the number of times for a specific outcome within n trials, each with probability of success, p, on a single trial;
Next, we define a threshold “t” as the minimal number of x that satisfies L >2 (i.e., the true signal needs to be 100 times or more than error.)
Hence, the sensitivity and “LoD” is given by:
Sensitivity = 1-pbinom(t-1,MEC,LoD),
Where “pbinom” is the cumulative probability of (t-1) or less successful trails from MEC binomial trials, each with LoD probability of success.

## Slide 6
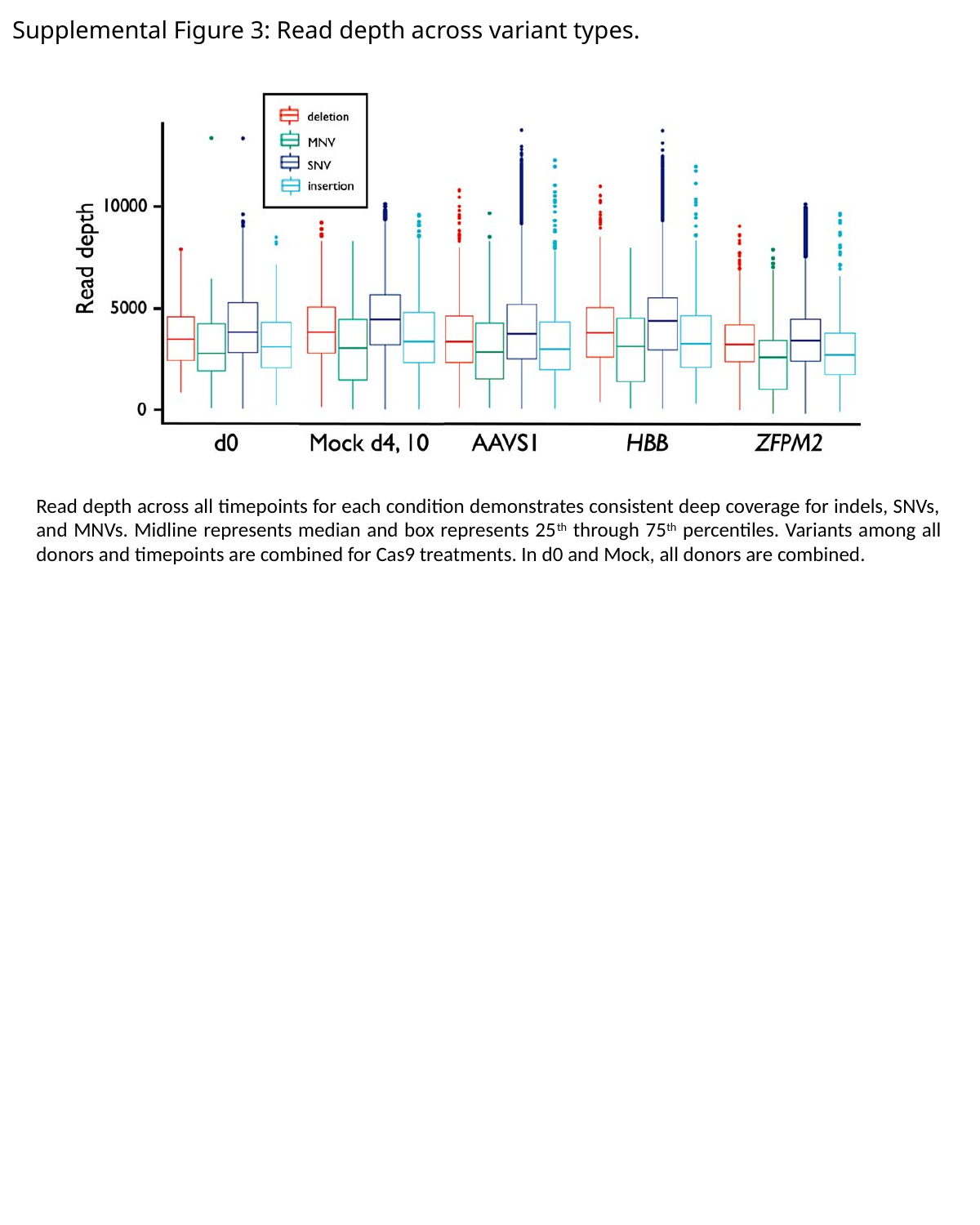

Supplemental Figure 3: Read depth across variant types.
Read depth across all timepoints for each condition demonstrates consistent deep coverage for indels, SNVs, and MNVs. Midline represents median and box represents 25th through 75th percentiles. Variants among all donors and timepoints are combined for Cas9 treatments. In d0 and Mock, all donors are combined.

## Slide 7
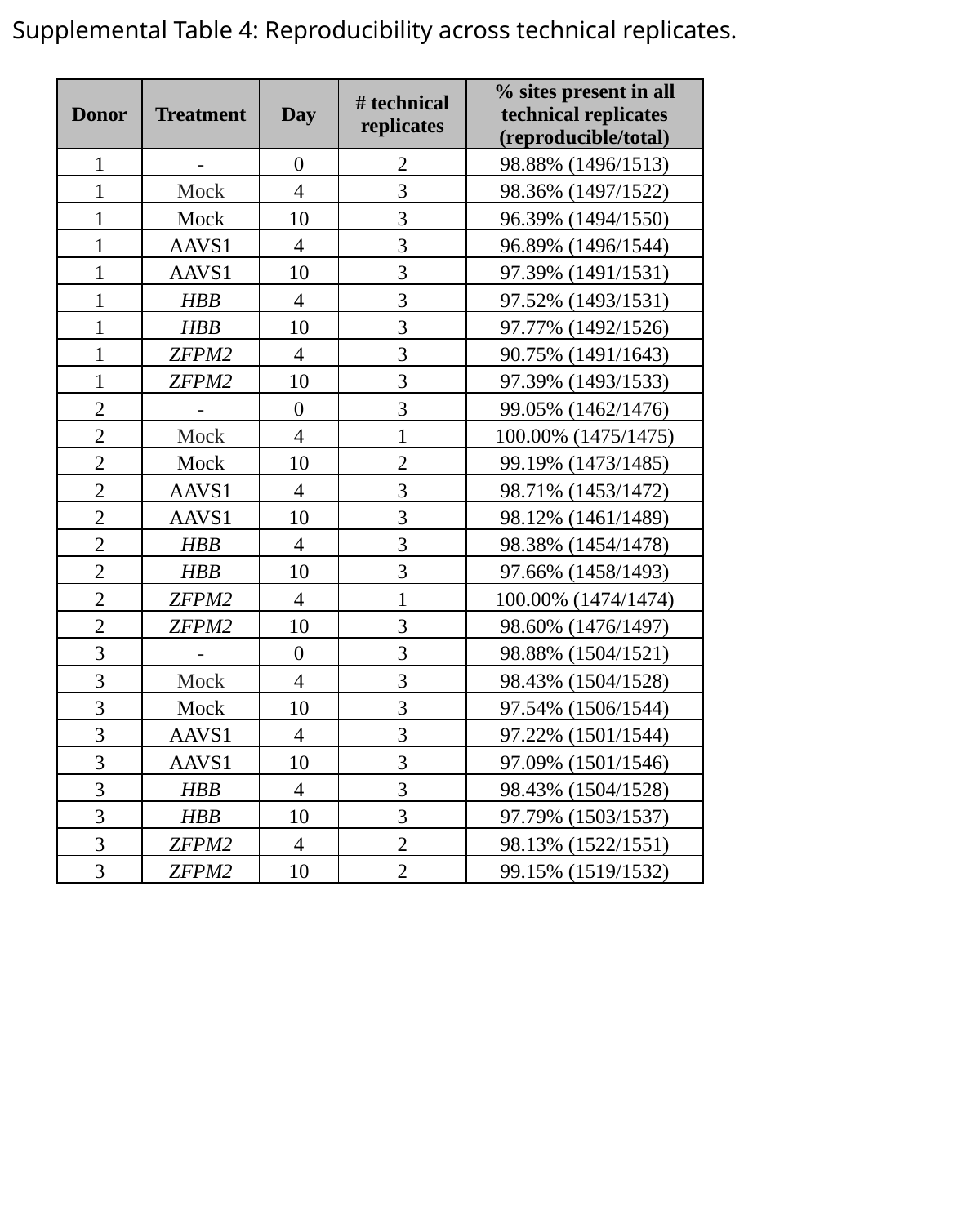

Supplemental Table 4: Reproducibility across technical replicates.
| Donor | Treatment | Day | # technical replicates | % sites present in all technical replicates (reproducible/total) |
| --- | --- | --- | --- | --- |
| 1 | - | 0 | 2 | 98.88% (1496/1513) |
| 1 | Mock | 4 | 3 | 98.36% (1497/1522) |
| 1 | Mock | 10 | 3 | 96.39% (1494/1550) |
| 1 | AAVS1 | 4 | 3 | 96.89% (1496/1544) |
| 1 | AAVS1 | 10 | 3 | 97.39% (1491/1531) |
| 1 | HBB | 4 | 3 | 97.52% (1493/1531) |
| 1 | HBB | 10 | 3 | 97.77% (1492/1526) |
| 1 | ZFPM2 | 4 | 3 | 90.75% (1491/1643) |
| 1 | ZFPM2 | 10 | 3 | 97.39% (1493/1533) |
| 2 | - | 0 | 3 | 99.05% (1462/1476) |
| 2 | Mock | 4 | 1 | 100.00% (1475/1475) |
| 2 | Mock | 10 | 2 | 99.19% (1473/1485) |
| 2 | AAVS1 | 4 | 3 | 98.71% (1453/1472) |
| 2 | AAVS1 | 10 | 3 | 98.12% (1461/1489) |
| 2 | HBB | 4 | 3 | 98.38% (1454/1478) |
| 2 | HBB | 10 | 3 | 97.66% (1458/1493) |
| 2 | ZFPM2 | 4 | 1 | 100.00% (1474/1474) |
| 2 | ZFPM2 | 10 | 3 | 98.60% (1476/1497) |
| 3 | - | 0 | 3 | 98.88% (1504/1521) |
| 3 | Mock | 4 | 3 | 98.43% (1504/1528) |
| 3 | Mock | 10 | 3 | 97.54% (1506/1544) |
| 3 | AAVS1 | 4 | 3 | 97.22% (1501/1544) |
| 3 | AAVS1 | 10 | 3 | 97.09% (1501/1546) |
| 3 | HBB | 4 | 3 | 98.43% (1504/1528) |
| 3 | HBB | 10 | 3 | 97.79% (1503/1537) |
| 3 | ZFPM2 | 4 | 2 | 98.13% (1522/1551) |
| 3 | ZFPM2 | 10 | 2 | 99.15% (1519/1532) |

## Slide 8
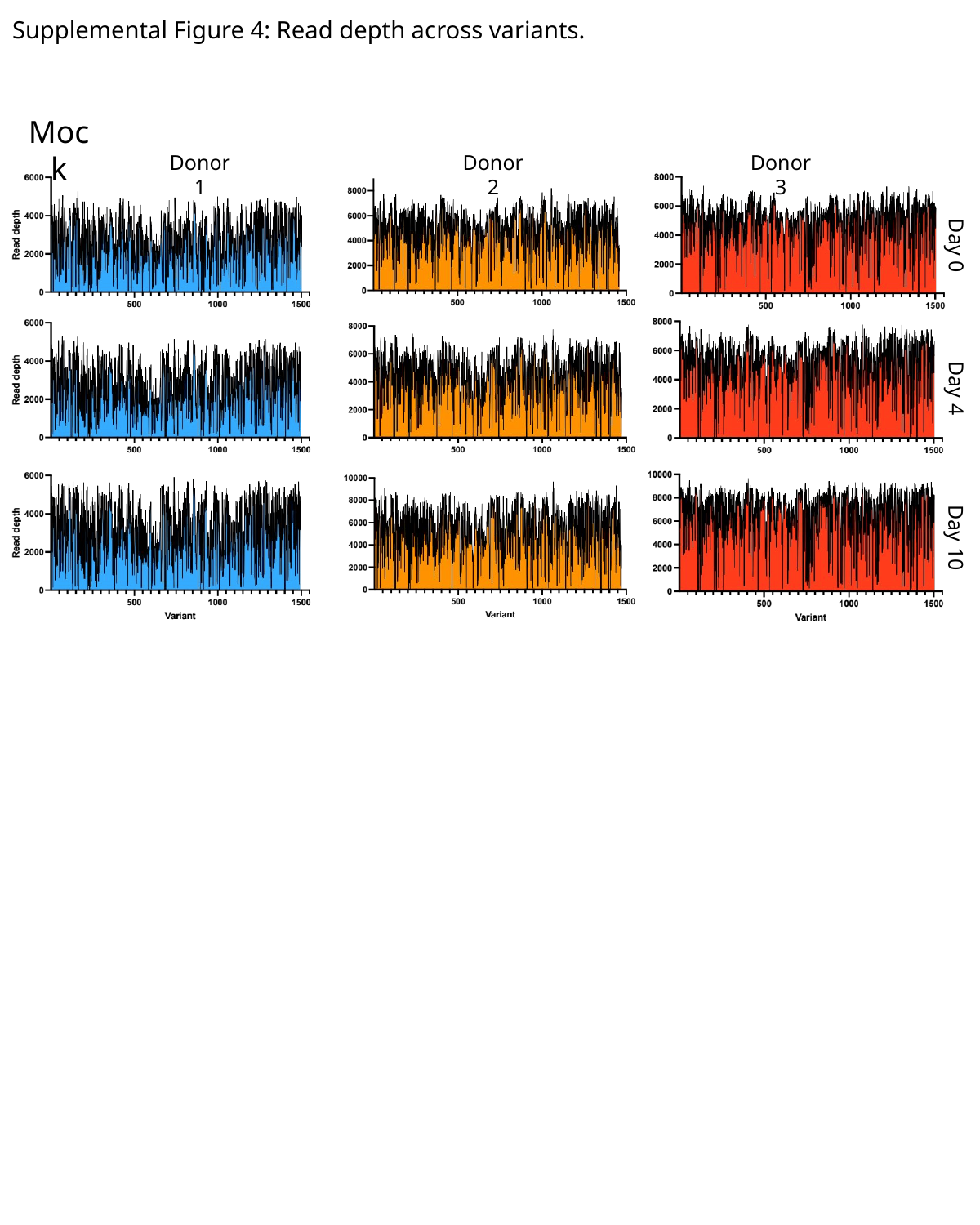

Supplemental Figure 4: Read depth across variants.
Mock
Donor 1
Donor 2
Donor 3
Day 0
Day 4
Day 10

## Slide 9
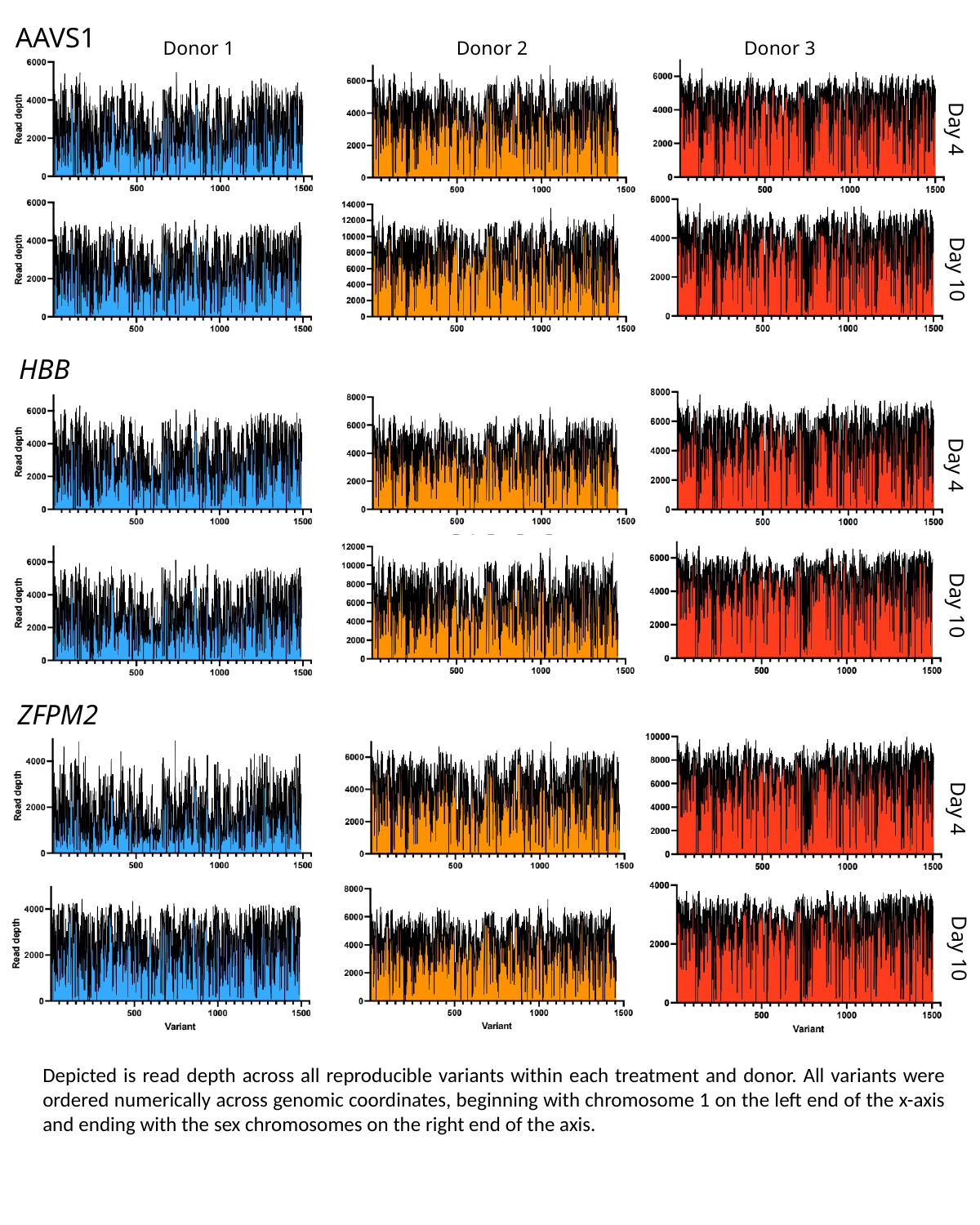

AAVS1
Donor 1
Donor 2
Donor 3
Day 4
Day 10
HBB
Day 4
Day 10
ZFPM2
Day 4
Day 10
Depicted is read depth across all reproducible variants within each treatment and donor. All variants were ordered numerically across genomic coordinates, beginning with chromosome 1 on the left end of the x-axis and ending with the sex chromosomes on the right end of the axis.

## Slide 10
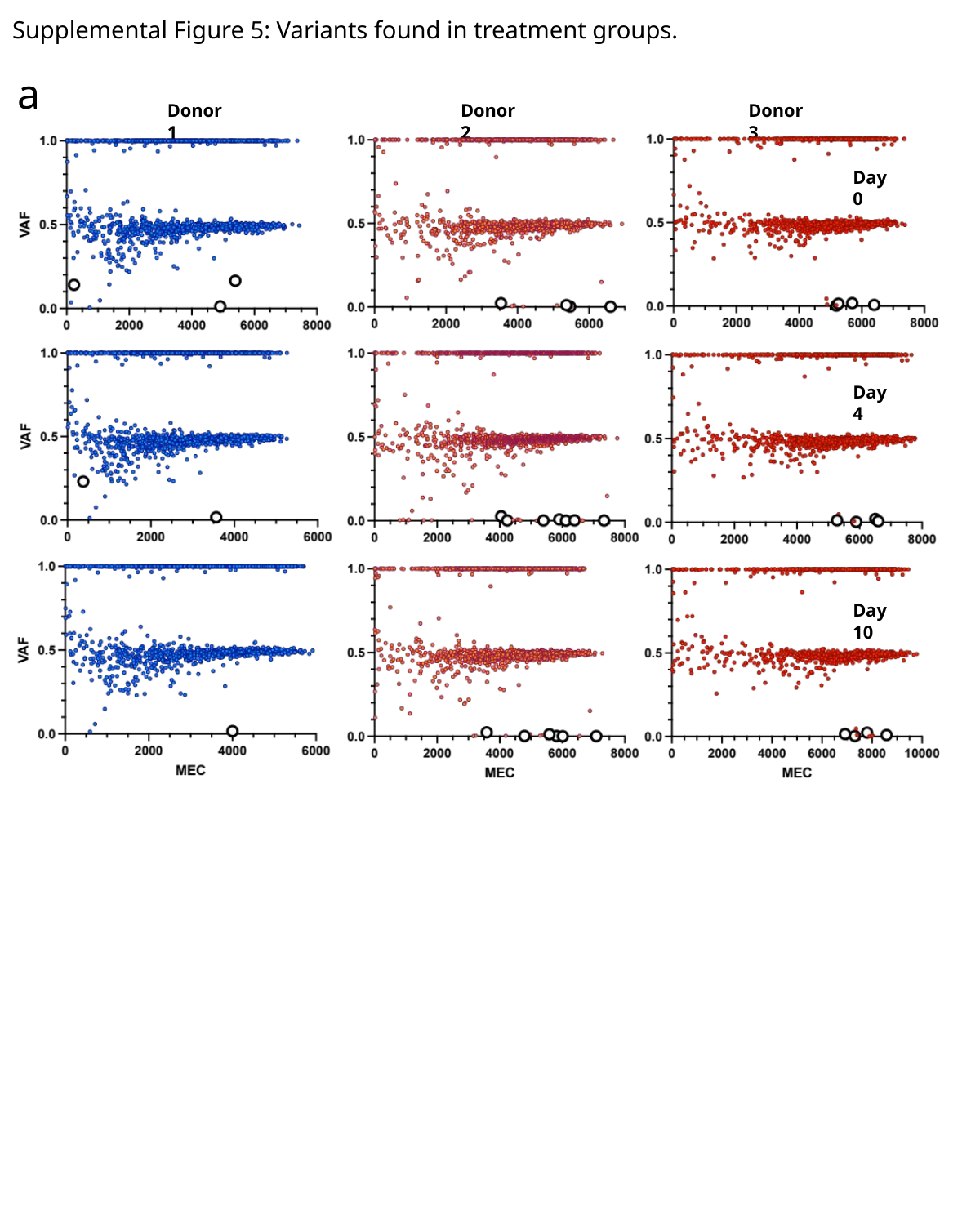

Supplemental Figure 5: Variants found in treatment groups.
a
Donor 1
Donor 2
Donor 3
Day 0
Day 4
Day 10

## Slide 11
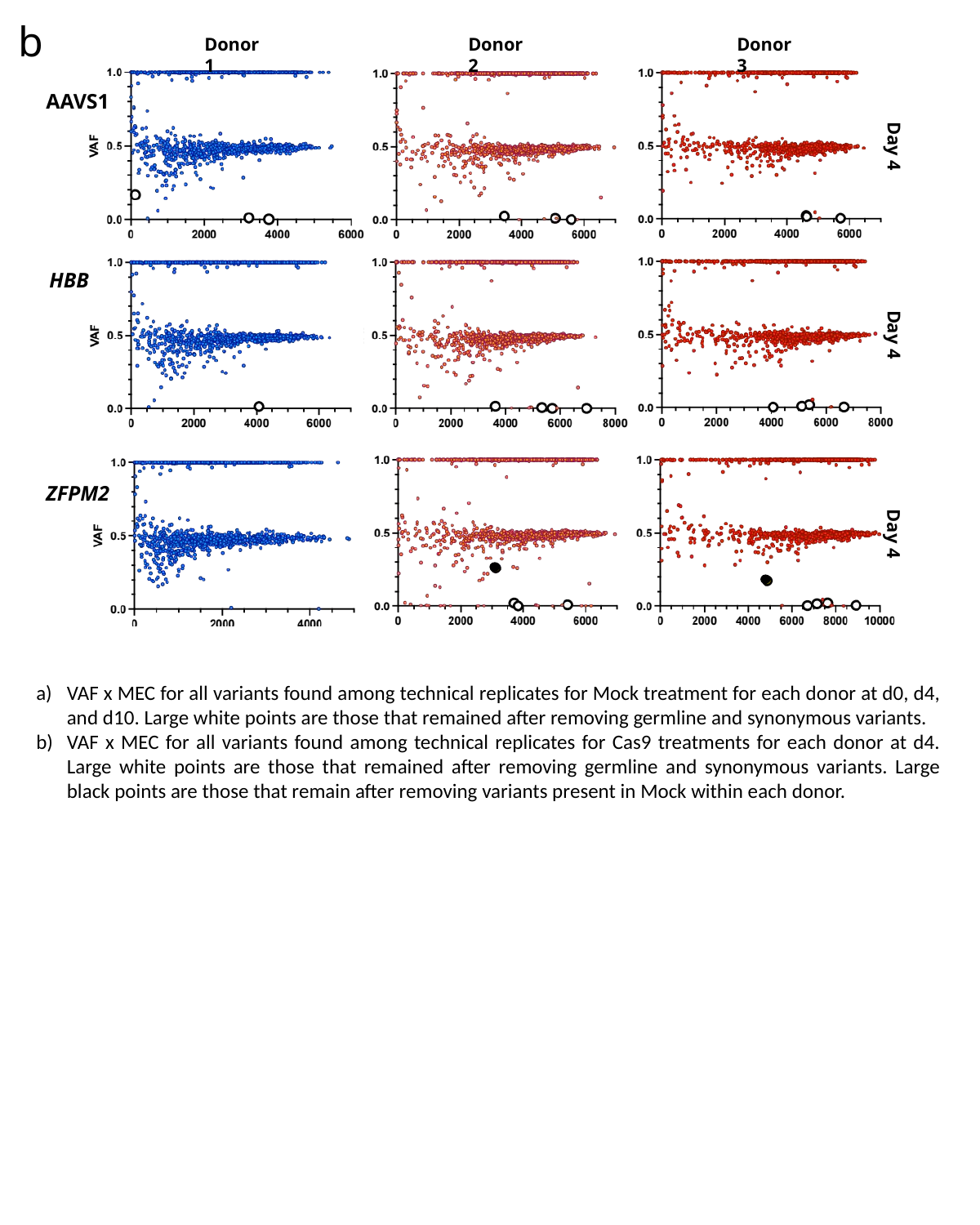

b
Donor 1
Donor 2
Donor 3
AAVS1
Day 4
HBB
Day 4
ZFPM2
Day 4
VAF x MEC for all variants found among technical replicates for Mock treatment for each donor at d0, d4, and d10. Large white points are those that remained after removing germline and synonymous variants.
VAF x MEC for all variants found among technical replicates for Cas9 treatments for each donor at d4. Large white points are those that remained after removing germline and synonymous variants. Large black points are those that remain after removing variants present in Mock within each donor.

## Slide 12
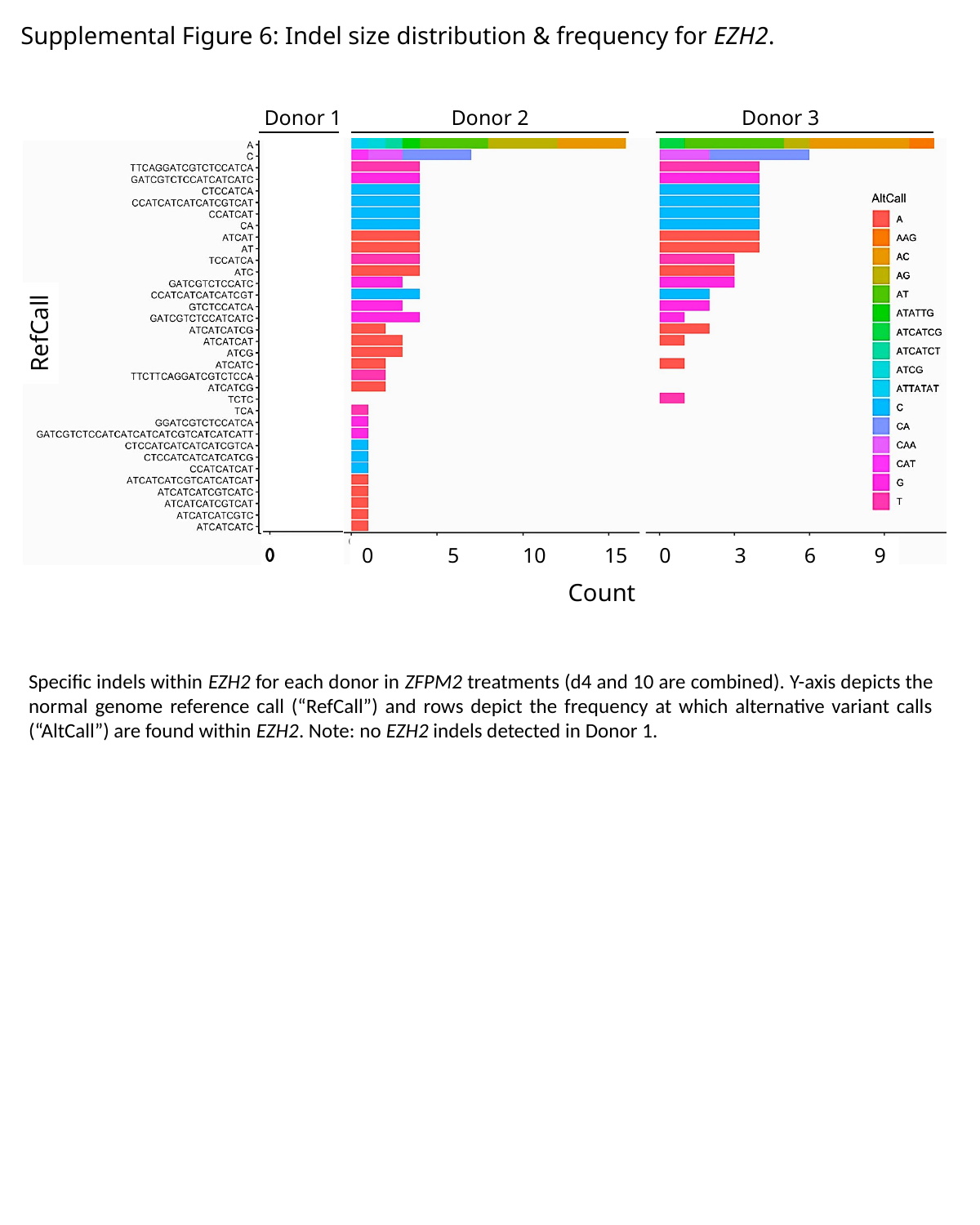

Supplemental Figure 6: Indel size distribution & frequency for EZH2.
Donor 1
Donor 2
Donor 3
RefCall
0 5 10 15 0 3 6 9
Count
Specific indels within EZH2 for each donor in ZFPM2 treatments (d4 and 10 are combined). Y-axis depicts the normal genome reference call (“RefCall”) and rows depict the frequency at which alternative variant calls (“AltCall”) are found within EZH2. Note: no EZH2 indels detected in Donor 1.

## Slide 13
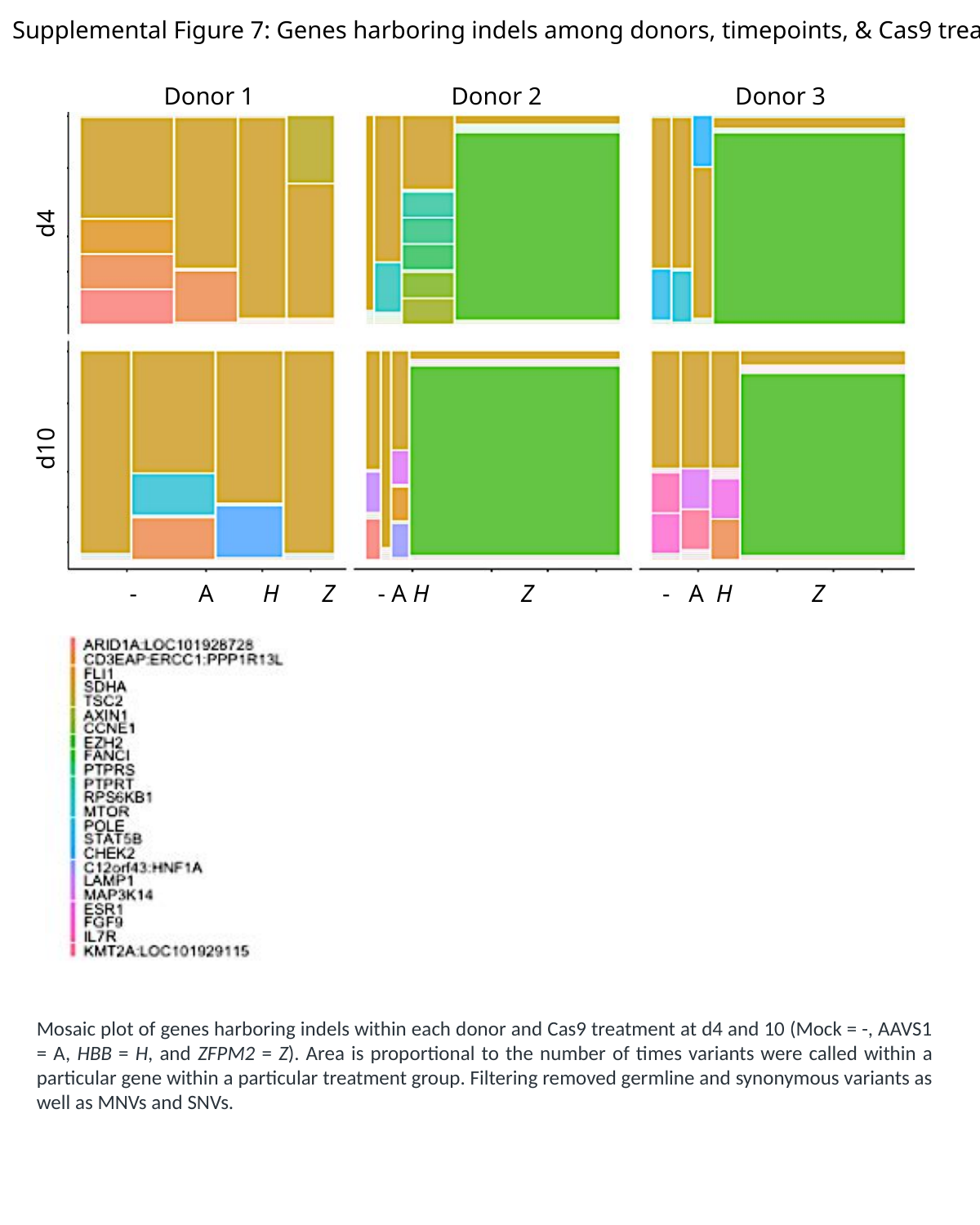

Supplemental Figure 7: Genes harboring indels among donors, timepoints, & Cas9 treatments.
Donor 1
Donor 2
Donor 3
d4
d10
- A H Z - A H Z - A H Z
Mosaic plot of genes harboring indels within each donor and Cas9 treatment at d4 and 10 (Mock = -, AAVS1 = A, HBB = H, and ZFPM2 = Z). Area is proportional to the number of times variants were called within a particular gene within a particular treatment group. Filtering removed germline and synonymous variants as well as MNVs and SNVs.

## Slide 14
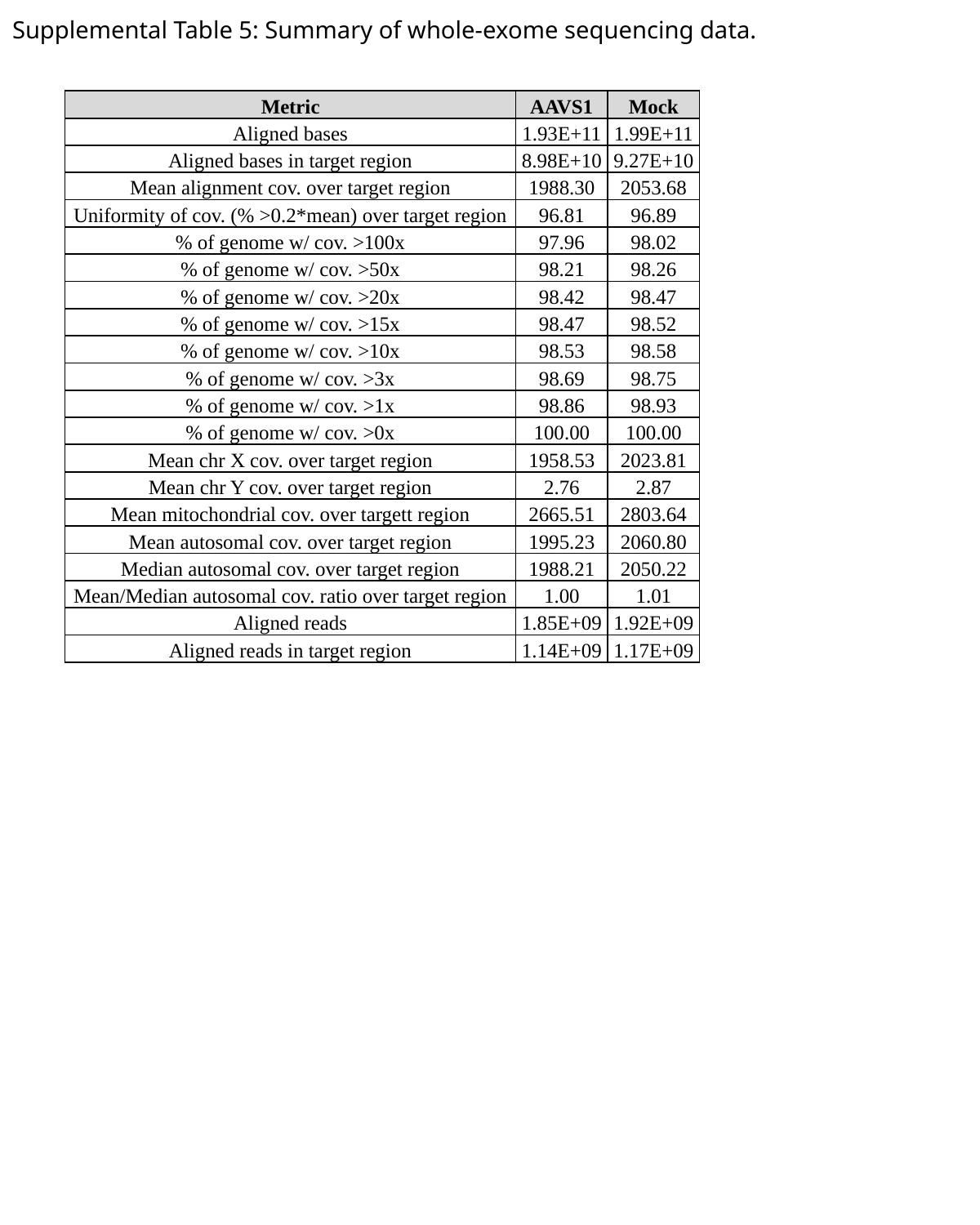

Supplemental Table 5: Summary of whole-exome sequencing data.
| Metric | AAVS1 | Mock |
| --- | --- | --- |
| Aligned bases | 1.93E+11 | 1.99E+11 |
| Aligned bases in target region | 8.98E+10 | 9.27E+10 |
| Mean alignment cov. over target region | 1988.30 | 2053.68 |
| Uniformity of cov. (% >0.2\*mean) over target region | 96.81 | 96.89 |
| % of genome w/ cov. >100x | 97.96 | 98.02 |
| % of genome w/ cov. >50x | 98.21 | 98.26 |
| % of genome w/ cov. >20x | 98.42 | 98.47 |
| % of genome w/ cov. >15x | 98.47 | 98.52 |
| % of genome w/ cov. >10x | 98.53 | 98.58 |
| % of genome w/ cov. >3x | 98.69 | 98.75 |
| % of genome w/ cov. >1x | 98.86 | 98.93 |
| % of genome w/ cov. >0x | 100.00 | 100.00 |
| Mean chr X cov. over target region | 1958.53 | 2023.81 |
| Mean chr Y cov. over target region | 2.76 | 2.87 |
| Mean mitochondrial cov. over targett region | 2665.51 | 2803.64 |
| Mean autosomal cov. over target region | 1995.23 | 2060.80 |
| Median autosomal cov. over target region | 1988.21 | 2050.22 |
| Mean/Median autosomal cov. ratio over target region | 1.00 | 1.01 |
| Aligned reads | 1.85E+09 | 1.92E+09 |
| Aligned reads in target region | 1.14E+09 | 1.17E+09 |

## Slide 15
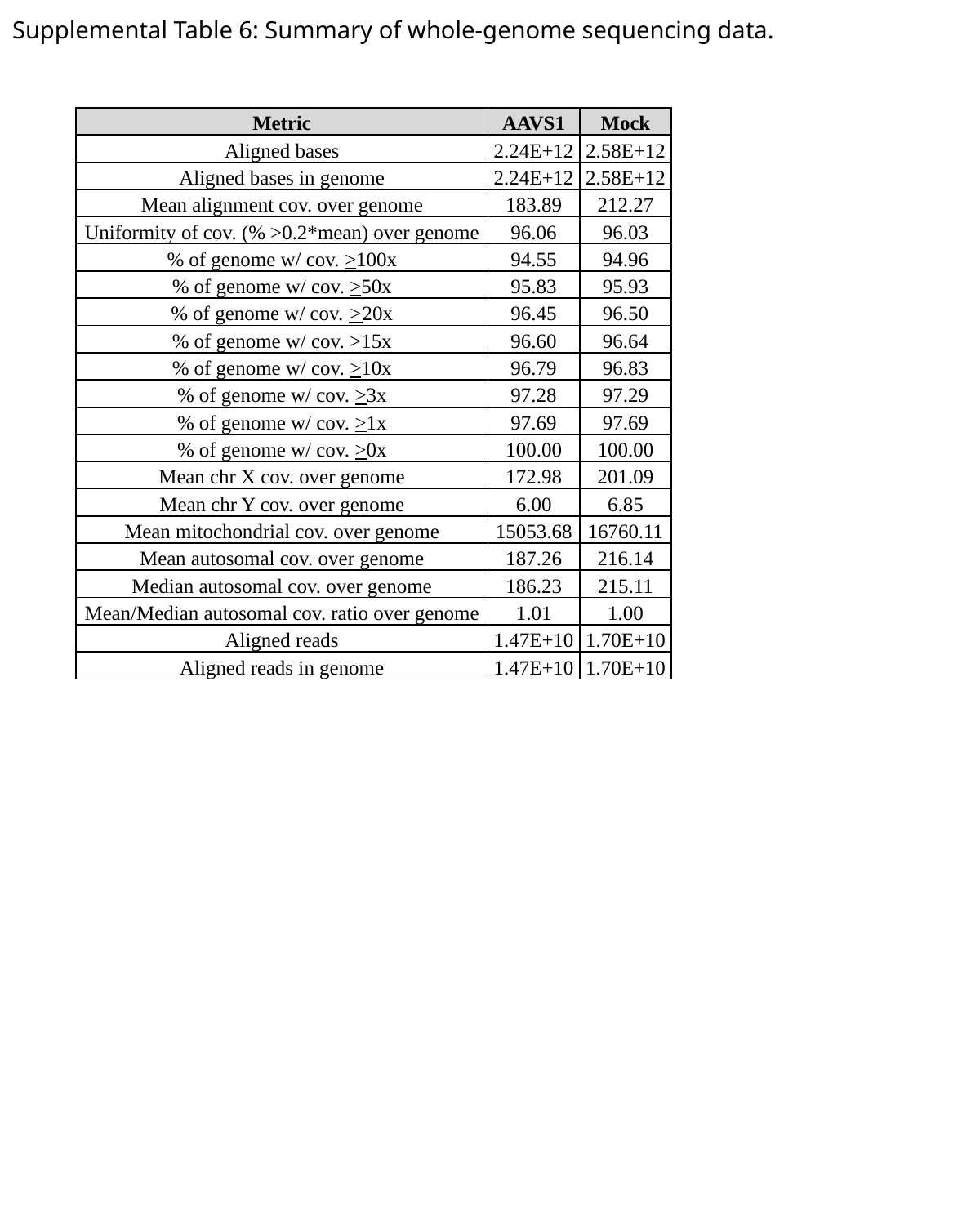

Supplemental Table 6: Summary of whole-genome sequencing data.
| Metric | AAVS1 | Mock |
| --- | --- | --- |
| Aligned bases | 2.24E+12 | 2.58E+12 |
| Aligned bases in genome | 2.24E+12 | 2.58E+12 |
| Mean alignment cov. over genome | 183.89 | 212.27 |
| Uniformity of cov. (% >0.2\*mean) over genome | 96.06 | 96.03 |
| % of genome w/ cov. >100x | 94.55 | 94.96 |
| % of genome w/ cov. >50x | 95.83 | 95.93 |
| % of genome w/ cov. >20x | 96.45 | 96.50 |
| % of genome w/ cov. >15x | 96.60 | 96.64 |
| % of genome w/ cov. >10x | 96.79 | 96.83 |
| % of genome w/ cov. >3x | 97.28 | 97.29 |
| % of genome w/ cov. >1x | 97.69 | 97.69 |
| % of genome w/ cov. >0x | 100.00 | 100.00 |
| Mean chr X cov. over genome | 172.98 | 201.09 |
| Mean chr Y cov. over genome | 6.00 | 6.85 |
| Mean mitochondrial cov. over genome | 15053.68 | 16760.11 |
| Mean autosomal cov. over genome | 187.26 | 216.14 |
| Median autosomal cov. over genome | 186.23 | 215.11 |
| Mean/Median autosomal cov. ratio over genome | 1.01 | 1.00 |
| Aligned reads | 1.47E+10 | 1.70E+10 |
| Aligned reads in genome | 1.47E+10 | 1.70E+10 |

## Slide 16
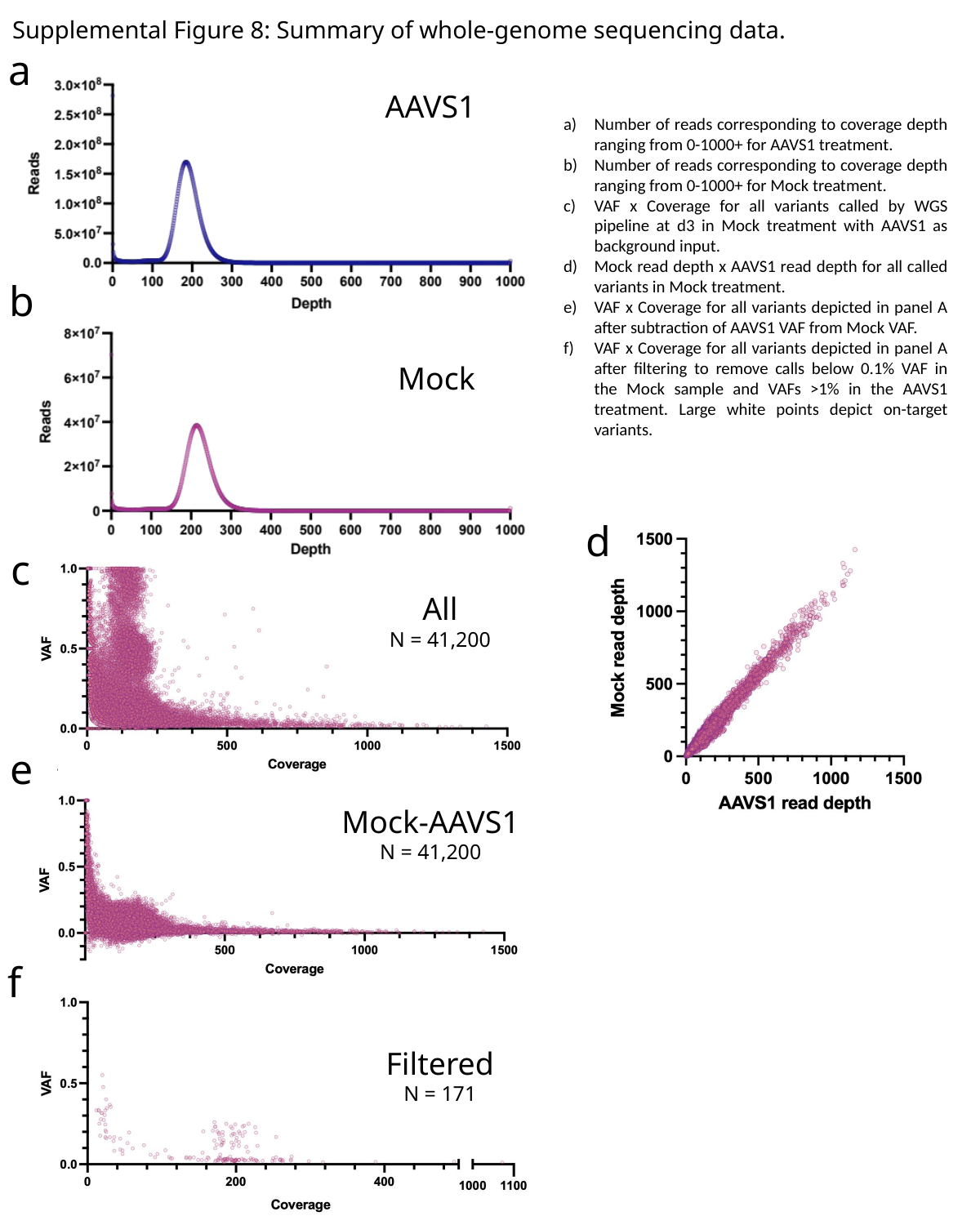

Supplemental Figure 8: Summary of whole-genome sequencing data.
a
AAVS1
Number of reads corresponding to coverage depth ranging from 0-1000+ for AAVS1 treatment.
Number of reads corresponding to coverage depth ranging from 0-1000+ for Mock treatment.
VAF x Coverage for all variants called by WGS pipeline at d3 in Mock treatment with AAVS1 as background input.
Mock read depth x AAVS1 read depth for all called variants in Mock treatment.
VAF x Coverage for all variants depicted in panel A after subtraction of AAVS1 VAF from Mock VAF.
VAF x Coverage for all variants depicted in panel A after filtering to remove calls below 0.1% VAF in the Mock sample and VAFs >1% in the AAVS1 treatment. Large white points depict on-target variants.
b
Mock
d
c
All
N = 41,200
e
Mock-AAVS1
N = 41,200
f
Filtered
N = 171
