# Supplementary material for: Ultra-deep sequencing validates safety of CRISPR/Cas9 genome editing in human hematopoietic stem and progenitor cells
Source: Nat Commun. 2022 Aug 11;13:4724. doi: 10.1038/s41467-022-32233-z (PMC9372057; doi:10.1038/s41467-022-32233-z)
Supplement: Supplementary file 2 — Reporting Summary [file 41467_2022_32233_MOESM2_ESM.pdf]

nature portfolio

Corresponding author(s): Matthew H. Porteus

Last updated by author(s): Apr 12, 2022

Reporting Summary

Nature Portfolio wishes to improve the reproducibility of the work that we publish. This form provides structure for consistency and transparency in reporting. For further information on Nature Portfolio policies, see our Editorial Policies and the Editorial Policy Checklist.

Statistics

For all statistical analyses, confirm that the following items are present in the figure legend, table legend, main text, or Methods section.

n/a	Confirmed	
☐	☒	The exact sample size (*n*) for each experimental group/condition, given as a discrete number and unit of measurement
☐	☒	A statement on whether measurements were taken from distinct samples or whether the same sample was measured repeatedly
☒	☐	The statistical test(s) used AND whether they are one- or two-sided<br>*Only common tests should be described solely by name; describe more complex techniques in the Methods section.*
☒	☐	A description of all covariates tested
☒	☐	A description of any assumptions or corrections, such as tests of normality and adjustment for multiple comparisons
☐	☒	A full description of the statistical parameters including central tendency (e.g. means) or other basic estimates (e.g. regression coefficient) AND variation (e.g. standard deviation) or associated estimates of uncertainty (e.g. confidence intervals)
☒	☐	For null hypothesis testing, the test statistic (e.g. *F*, *t*, *r*) with confidence intervals, effect sizes, degrees of freedom and *P* value noted<br>*Give P values as exact values whenever suitable.*
☒	☐	For Bayesian analysis, information on the choice of priors and Markov chain Monte Carlo settings
☒	☐	For hierarchical and complex designs, identification of the appropriate level for tests and full reporting of outcomes
☒	☐	Estimates of effect sizes (e.g. Cohen's *d*, Pearson's *r*), indicating how they were calculated

Our web collection on statistics for biologists contains articles on many of the points above.

Software and code

Policy information about availability of computer code

Data collection	DNA QC data was collected by Qubit dsDNA HS assay kit on a Qubit fluorometer as well as by an Agilent Technologies 2100 Bioanalyzer with a High Sensitivity DNA chip. All data from NGS library preps was generated using on the Illumina NovaSeq platform.
Data analysis	NGS raw data was processed using the DRAGEN v3.8.4 Enrichment pipeline. TIDE software was used to analyze indel formation data. Prism was used to plot most data presented in Main and Supplemental Figures.Prism version 9, TIDE version 3.3.0, RStudio Version 1.2.5033

For manuscripts utilizing custom algorithms or software that are central to the research but not yet described in published literature, software must be made available to editors and reviewers. We strongly encourage code deposition in a community repository (e.g. GitHub). See the Nature Portfolio guidelines for submitting code & software for further information.

Data

Policy information about availability of data

All manuscripts must include a data availability statement. This statement should provide the following information, where applicable:

- Accession codes, unique identifiers, or web links for publicly available datasets
- A description of any restrictions on data availability
- For clinical datasets or third party data, please ensure that the statement adheres to our policy

All high-throughput sequencing data will be uploaded to a public repository available on Dryad (https://dryad.org/stash).

Field-specific reporting

Please select the one below that is the best fit for your research. If you are not sure, read the appropriate sections before making your selection.

☒ Life sciences ☐ Behavioural & social sciences ☐ Ecological, evolutionary & environmental sciences

For a reference copy of the document with all sections, see nature.com/documents/nr-reporting-summary-flat.pdf

Life sciences study design

All studies must disclose on these points even when the disclosure is negative.

Sample size	Sample sizes of a minimum of three or more CD34+ HSPC donors were chosen due to the degree of variation observed across separate donors in prior Cas9-mediated genome editing studies (Dever, et al. Nature 2016; Gomez-Ospina, et al. Nature Commun 2019; & Pavel-Dinu, et al. Nature Commun 2019).
Data exclusions	No data exclusion criteria were established prior to the execution of any experiments reported in this paper, and no data were excluded following conclusion of the experiments.
Replication	All experiments were performed across a minimum of three or more CD34+ HSPC donors. All technical replicates, where sufficient input material was available, were successful.
Randomization	No treatment randomization or exclusion criteria during data collection or analysis was necessary during the course of the experiments reported in this study, which is in agreement with prior similar studies (Dever et at. Nature 2016; Pavel-Dinu et at. Nat Commun 2019; Gomez-Ospina et at. Nat Commun 2019). Treatment conditions were blinded to the analytics teams since all donors were subject to positive and negative treatments such that further randomization was unnecessary..
Blinding	In order to minimize any unintended bias, the treatment conditions for library-prepped samples were blinded to the Illumina collaborators who ran these on the Illumina NGS platform and then processed initial raw data.

Reporting for specific materials, systems and methods

We require information from authors about some types of materials, experimental systems and methods used in many studies. Here, indicate whether each material, system or method listed is relevant to your study. If you are not sure if a list item applies to your research, read the appropriate section before selecting a response.

Materials & experimental systems

n/a	Involved in the study	
☒	☐	Antibodies
☒	☐	Eukaryotic cell lines
☒	☐	Palaeontology and archaeology
☒	☐	Animals and other organisms
☐	☒	Human research participants
☒	☐	Clinical data
☒	☐	Dual use research of concern

Methods

n/a	Involved in the study	
☒	☐	ChIP-seq
☒	☐	Flow cytometry
☒	☐	MRI-based neuroimaging

Human research participants

Policy information about studies involving human research participants

Population characteristics	Human-derived samples were de-identified prior to use in our studies and we therefore had no access to characteristics of patient samples used in this study.
Recruitment	Participants were recruited by Stanford's Binns Family Cord Blood Program. Selection bias in this case would result from the population of patients who have access to care at Stanford Hospital. However, we believe that selection bias is accounted for due to the fact that each patient-derived sample was subdivided and then subjected to various treatment conditions, thereby accounting for any donor-to-donor variability inherent to the samples.
Ethics oversight	Informed patient consent was acquired and patients were recruited in accordance with Stanford IRB protocol number 33813.

Note that full information on the approval of the study protocol must also be provided in the manuscript.

Stanford University Institutional Review Board can be reached at the research compliance office Stanford University https://researchcompliance.stanford.edu/panels/hs/about/contacts
